# Clinical Presentation and Outcomes of Myocarditis Post mRNA Vaccination: A Meta-Analysis and Systematic Review

**DOI:** 10.7759/cureus.19240

**Published:** 2021-11-03

**Authors:** Abhishek Matta, Rajesh Kunadharaju, Marcus Osman, Christy Jesme, Zachary McMiller, Erika M Johnson, Danielle Matta, Rekha Kallamadi, Dinesh Bande

**Affiliations:** 1 Internal Medicine, Sanford Health, Fargo, USA; 2 Internal Medicine, University of North Dakota School of Medicine and Health Sciences, Fargo, USA; 3 Department of Pulmonary and Critical Care Medicine, University of Buffalo, Buffalo, USA; 4 Internal Medicine, University of North Dakota School of Medicine, Fargo, USA

**Keywords:** moderna, pfizer-biontech, mrna-based vaccine, post vaccination myocarditis, myocarditis

## Abstract

Background: Myocarditis is being increasingly reported as a potential complication of both Pfizer-BioNTech and Moderna vaccines for COVID-19. One thousand five hundred and twenty-two cases were reported as of September 02, 2021, as per CDC’s (Centers for Disease Control) vaccine adverse event reporting system. Most of the published data is available in the form of case reports and series. There is a need to compile the demographic data, clinical features, and outcomes in these patients.

Methods: A systematic search was conducted in PubMed, Embase, Web of science, and google scholar for published literature between January 01, 2020, and July 17, 2021. Individual data of 69 patients were pooled from 25 qualifying case reports and case series.

Results: The median age of onset was 21 years. 92.7% of the patients were male. 76.8% of patients received the Pfizer-BioNTech vaccine, and 23.2% received the Moderna vaccine. 88.5% developed symptoms after the second dose. Patients were admitted to the hospital a median of three days post-vaccination. All the patients had chest pain and elevated troponin. The myocarditis was confirmed on cardiac MRI in 87% of the patients. Most of the patients had late gadolinium enhancement on MRI. The median length of stay was four days. All the reported patients recovered and were discharged.

Conclusion: Post-mRNA vaccination myocarditis is seen predominantly in young males within a few days after their second dose of vaccination. The pathophysiology of myocarditis is not well known. The prognosis is good as all the reported patients recovered. The presence of late gadolinium enhancement on cardiac MRI indicated myocardial necrosis/fibrosis and further studies are needed to establish the long-term prognosis of the condition.

## Introduction and background

The United States Food and Drug Administration (FDA) issued an emergency use authorization (EUA) for the Pfizer-BioNTech mRNA vaccine (BNT162b2) on December 11, 2020, and for the Moderna mRNA vaccine (mRNA-1273) on December 18, 2020 [1]. Neither of the initial studies by Baden et al. and Polack et al. established the efficacy of mRNA vaccines reported any myocarditis [2,3]. Initial reports of myocarditis post mRNA vaccination were reported from Israel in April 2021 [4].

According to the Center for Disease Control (CDC) vaccine adverse event reporting system (VAERS), as of September 02, 2021, there have been 1522 reports of myocarditis after COVID-19 vaccination [5]. 1013 cases were reported following Pfizer-BioNTech vaccine, 475 following Moderna vaccine, and 31 following Janssen vaccine [5]. Most of the published clinical information on post-mRNA vaccination myocarditis is available in the form of case reports and case series.

The goal of this systematic review is to compile the available individual patient data from the published case reports and series to assess the demographics, risk factors, clinical presentation, investigational findings including electrocardiogram (EKG), echocardiogram (Echo) and cardiac magnetic resonance imaging (MRI), hospital length of stay (LOS), and outcomes in patients who developed myocarditis following administration of mRNA vaccine for COVID-19 to enable clinicians to accurately recognize, evaluate, and manage the condition.

## Review

Methods

**Figure 1:**
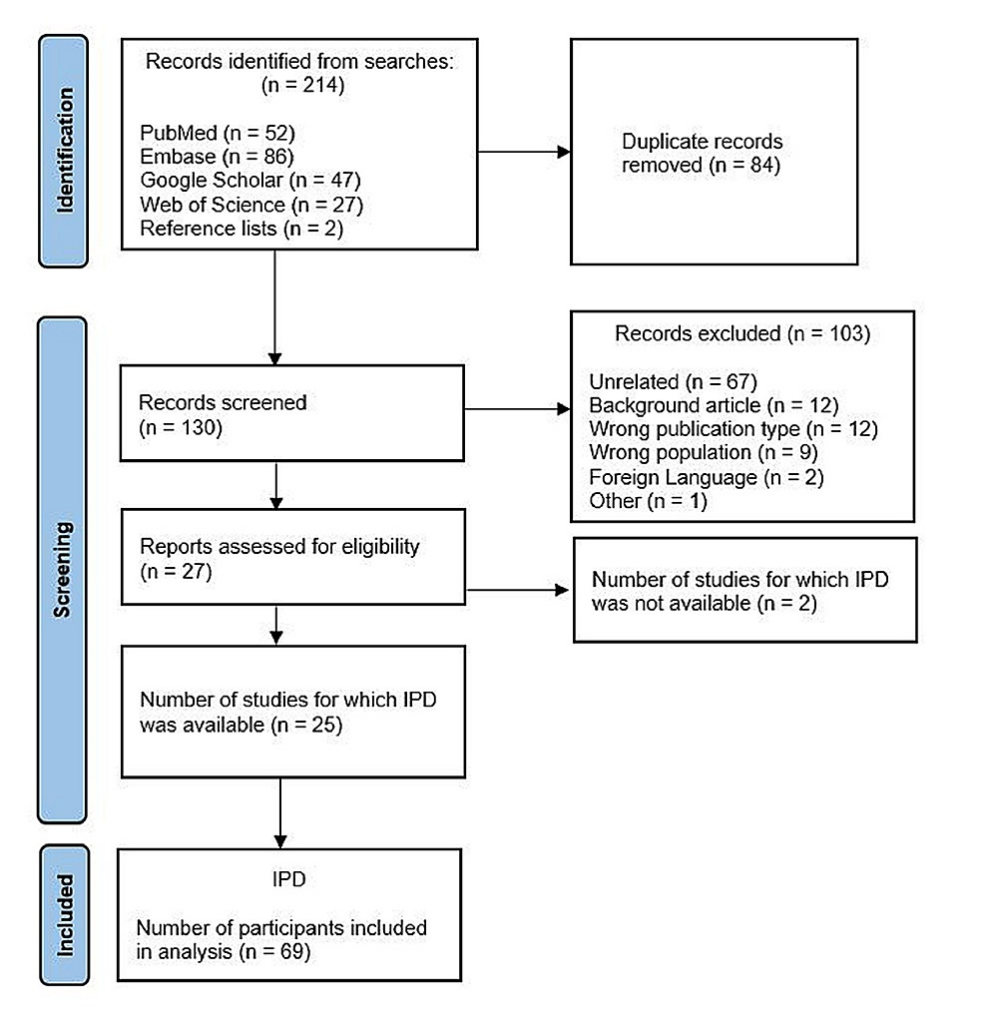
Prisma flow diagram

Literature was searched in PubMed, Embase, Google Scholar, and Web of Science from January 1, 2020, to July 17, 2021, for case reports and case series of myocarditis post mRNA vaccination. Search strategies will be provided on request. Hundred and twenty-eight unique articles were obtained. The articles were compiled in Rayyan software for review. Each manuscript was reviewed by at least two reviewers to identify case reports and case series of patients who developed myocarditis after vaccination with either Pfizer-BioNTech or Moderna mRNA vaccine. Case reports and case series published in English reporting detailed individual patient demographic data of patients hospitalized for post-mRNA vaccination myocarditis were included in our review. All other publication types and articles published in languages other than English were excluded. Twenty-five unique manuscripts which reported post-mRNA vaccination myocarditis were identified [1,6-29]. Reference search resulted in the identification of two more articles [30,31]. Individual demographic data were not available in two studies, and they were excluded from the pooled data analysis [16,29]. The PRISMA flow diagram is available in Figure 1.

Conflicts about inclusion criteria were resolved with group discussion. The included articles were reviewed in detail by at least two reviewers for individual patient data including demographics, medical history, clinical symptoms, laboratory data, EKG, echo, cardiac MRI findings, treatment received, LOS, outcomes, and they were tabulated in an Excel spreadsheet. One patient from Rosner et al. was excluded as the patient received Johnson and Johnson vaccine [10]. Individual data from 69 patients in the 25 articles were pooled for the final analysis. Findings are reported following the PRISMA statement in health care interventions. The list of included articles along with the quality of evidence ratings modified from the Oxford Centre for Evidence-Based Medicine [32], age, sex distribution, vaccine type, days to hospitalization, clinical symptoms, EKG, MRI findings, treatment administered, and LOS from individual studies are reported in Table 1.

**Table 1:** List of included manuscripts with data *Quality of evidence ratings modified from the Oxford Centre for Evidence-based Medicine (4=case series, 5=case report). ^#^Number is as reported for case report and reported as the median for case series. NSAID: nonsteroidal anti-inflammatory drug, IVIG: intravenous immunoglobulin.

Author	Quality of evidence*	Number of patients	Male (%)	Age (years)^#^	Symptoms after the second dose (%)	Hospital Presentation (the day after vaccination)^#^	Symptoms reported	ST-elevation on EKG (%)	Abnormal MRI (%)	Anti-inflammatory treatment used	Hospital LOS (days)^#^
Deb et al. [24]	5	1	100	67	100	1	Fever, chills, fatigue, nausea, orthopnea, dyspnea	0	Not reported	None reported	2
Habib et al. [23]	5	1	100	37	100	3	Chest pain, generalized body aches, fever, chills, headache	100	100	None reported	6
Garcia et al. [28]	5	1	100	39	100	1	Fever, chest pain	100	100	NSAIDs	6
Nevet [22]	4	3	100	24	100	2	Fever, chest pain	100	100	NSAIDs, Colchicine	Not reported
Ammirati et al. [27]	5	1	100	56	100	3	Chest pain	100	100	None reported	7
Marshall et al. [26]	4	7	100	17	100	3	Chest pain, nausea, fatigue, vomiting, headache, anorexia, fever, chills, malaise, dyspnea, sore throat, cough, body aches	100	100	NSAIDs, Colchicine, IVIG, steroids	3
D'Angelo et al. [17]	5	1	100	30	100	3	Fever, nausea, arthralgias, chest pain, dyspnea, sweating	100	100	NSAIDs, steroids	7
Muthukumar et al. [20]	5	1	M	52	100	3	Fever, myalgia, headache, chest pain	0	100	None reported	4
Larson et al. [21]	4	8	100	28.5	88	3	Fever, chills, myalgia, chest pain, dyspnea, cough	75	100	NSAIDs, steroids, Colchicine	Not reported
Mansour et al. [1]	4	2	50	23	100	1.5	Fever, chills, chest pain, lightheadedness	100	100	None reported	2.5
Albert et al. [18]	5	1	100	24	100	4	Fever, chills, myalgias, chest pain	0	100	None reported	Not reported
Abu Mouch et al. [25]	4	6	100	22	83	2.5	Chest pain	100	100	NSAIDs, Colchicine	6
Kim et al. [19]	4	4	75	30	100	2.5	Fever, fatigue, myalgia, chest pain, dyspnea, diaphoresis, palpitation, headache, injection site pain	100	100	NSAIDs, Colchicine, Steroids	2-4 days
Rosner et al. [10]	4	6	100	23.5	83	3	Chest pain, dyspnea, fever, chills, myalgia, headache	33	100	NSAIDs, Colchicine, Steroids	3
Singh et al. [7]	5	1	100	24	100	3	Chest pain, headache	0	100	None reported	4
Vidula et al. [14]	4	2	100	18.5	100	2.5	Chest pain, dyspnea, fever, myalgia	100	100	NSAIDs, Colchicine	Not reported
Starekova et al. [11]	4	5	80	21	100	3	Chills, headache, fever, chest pain, dyspnea, nausea, myalgia, fatigue, lightheadedness, malaise	40	100	None reported	Not reported
Tano et al. [12]	5	1	100	29	0	6	Fever, chest pain	100	100	NSAIDs, Colchicine	9
Watkins et al. [13]	5	1	M	20	100	2	Chest pain, dyspnea	100	100	NSAIDs, Colchicine	Not reported
Shaw et al. [9]	4	4	50	20.5	50	4	Chest pain/pressure	25	100	None reported	Not reported
Park et al. [8]	4	2	100	15.5	50	2.5	Chest pain	100	Not reported	IVIG	4
Cereda et al. [15]	5	1	100	21	100	1	Chest pain	100	100	None reported	7
Minocha et al. [6]	5	1	100	17	100	2	Fever, body aches, chest pain	100	100	NSAIDs	6
McLean and Johnson [31]	5	1	100	16	100	3	Fever, body aches, chest pain	100	100	IVIG, NSAIDs	6

We performed a pooled data analysis where continuous variables were found to be in nonuniform distribution and therefore reported as median and range. The rest of the pooled data was reported as frequency and proportion. The complete pooled data from the 25 studies are reported in Table 2.

**Table 2:** Summary of pooled data from 25 included articles OSA: obstructive sleep apnea, COPD: chronic obstructive pulmonary disease, CRP-C: reactive protein, ESR: erythrocyte sedimentation rate, BNP: brain natriuretic peptide, CPK: creatinine phosphokinase, EF: ejection fraction, LGE: late gadolinium enhancement, NSAID: nonsteroidal anti-inflammatory drug, IVIG: intravenous immunoglobulin.

Summary of pooled data from included studies	
Age	Total patients - 69
	Median - 21 years (range 14–70 years)
Sex (n)%	Male - 64 (92.7%)
	Female - 5 (7.3%)
Vaccine (n)%	Pfizer-BioNTech - 53 (76.8%)
	Moderna - 16 (23.2%)
Past medical history (n)%	No significant history reported - 56 (81.2%)
	Post COVID - 6 (8.7%)
	Hypertension - 4 (5.8%)
	Hyperlipidemia - 4 (5.8%)
	Hyperlipidemia - 4 (5.8%)
	Tobacco use - 4 (5.8%)
	Hypothyroidism - 2 (2.9%)
	Myocarditis - 1 (1.4%)
	Congestive heart failure - 1 (1.4%)
	Atrial fibrillation - 1 (1.4%)
	Diabetes mellitus - 1 (1.4%)
	OSA - 1 (1.4%)
	Asthma - 1 (1.4%)
	COPD - 1 (1.4%)
Symptom onset after vaccination	Median - 2 days (range 0–25 days)
	After first dose (n=8): median 4 days (range 2–25 days)
	After second dose (n=61): median 2 days (range 0–4 days)
Days to hospitalization after vaccination	Median - 3 days (range 1–25 days)
Symptoms (n)%	Chest pain/pressure - 69 (100%)
	Fevers - 31 (44.9%)
	Myalgia/body aches - 16 (23.2%)
	Chills - 15 (21.7%)
	Dyspnea - 13 (18.8%)
	Headache - 10 (14.5%)
	Fatigue - 8 (11.6%)
	Nausea/vomiting - 6 (8.7%)
	Syncope - 2 (2.9%)
	Lightheadedness - 2 (2.9%)
	Diarrhea - 1 (1.5%)
Highest reported value of troponin	Troponin I (24) - median 8.161 ng/mL (range 0.37–44.8 ng/mL)
	Troponin T (19) - median 1.332 ng/mL (range 0.39– 3.72 ng/mL)
	High sensitivity troponin T (9) - median 0.70 ng/mL (range 0.18–15.34 ng/mL)
	High sensitivity troponin I (4) - median 6.90 ng/mL (range 6.77–14.35 ng/mL)
	Troponin reported as multiple of upper limit of normal (8). Median 192.5 (range 29–1433).
	Troponin not specified (1) - reported value 0.11 ng/mL
	High-sensitivity troponin not specified (1) - reported value 32.14 ng/mL
	Reported as elevated (3)
Elevated inflammatory and other cardiac biomarkers (n)%	Reported - 52/69 patients (75.4%)
	CRP - 48 (92%)
	ESR - 15 (23%)
	BNP/Pro-BNP - 8 (15%)
	D-dimer - 4 (7.7%)
	CPK - 3 (5.7%)
EKG (n)%	Reported - 66/69 patients (95.7%)
	ST elevation - 52 (78.8%)
	Normal - 5 (7.5%)
	Nonspecific ST-T wave abnormalities - 5 (7.5%)
	PR depression - 2 (3%)
	ST depression - 1 (1.5%)
	Peak T waves - 1 (1.5%)
	Left axis deviation with incomplete RBBB - 1 (1.5%)
	AV dissociation with junctional escape rhythm - 1 (1.5%)
Echocardiography (n)%	Reported - 55/69 (79.7%)
	EF > 50% with no regional wall abnormalities - 37 (67.3%)
	Hypokinesis - 16 (29.1%)
	EF < 50% - 5 (9.1%)
	Pericardial effusion - 2 (3.6%)
Cardiac MRI (n)%	Reported - 60/69 patients (87%)
	Positive for myocarditis - 60 (100%)
	LGE reported - 53 (88.3%)
Treatment (n)%	Specific anti-inflammatory therapy reported in 42/69 patients (60.9%)
	NSAIDS - 36 (85.7%)
	Colchicine - 21 (50%)
	Steroids - 9 (21.4%)
	IVIG - 6 (14.3%)
Length of hospital stay	Reported: 41/69 patients (59.4%)
	Median 4 days (range 2-9 days)
Recovery	Reported: 60/69 patients (87%)
	100% recovery rate

The study protocol is submitted to but not registered yet at PROSPERO. A list of excluded articles is available on request. The data from 25 of the 27 articles on the condition were used for the pooled analysis and the results from the remaining two studies were mentioned in the discussion to avoid publication bias. Reporting bias exists as not all data parameters were reported uniformly in every study. Each parameter was reported as a proportion of reported cases to mitigate reporting bias.

Results

Demographics

For the 69 patients included in our study, the median age was 21 years, ranging from 14 to 70 years; 92.7% were male, and 7.3% were female. 76.8% of patients received the Pfizer-BioNTech vaccine, and the rest of the 23.2% received the Moderna vaccine. 11.5% of the patients developed symptoms after the first dose, and 88.5% developed symptoms after the second dose. 81.2% did not report any significant past medical history. 8.7% of included patients had prior COVID-19 infection. Among the patients with a history of COVID-19 infection, 50% developed myocarditis after the first dose of the vaccine.

Clinical Presentation

The median onset of symptoms was two days post-vaccination (range 0-25 days). The median duration from vaccination to hospitalization was three days (range 1-25 days). The predominant symptoms reported are chest pain/pressure (100%), fever (44.9%), myalgia/body ache (23.2%), chills (21.7%), and dyspnea (18.8%). Other symptoms reported at a lesser frequency include headache, nausea, vomiting, fatigue, lightheadedness, syncope, and diarrhea.

Assessment and Diagnosis

Cardiac biomarkers: All the patients included in this review reported an elevation in troponin during the acute presentation. The type of troponin used was heterogenous between all studies. Troponin I was commonly performed and reported in 35% of patients and the median of highest reported values was 8.2 ng/mL (range 0.37-44.8 ng/mL). Troponin T was reported in 27.5% of patients and the median of highest reported values was 1.3 ng/mL (range 0.39-13.72 ng/mL). High sensitivity Troponin T was reported in 13% of patients and the median of highest reported values was 0.70 ng/mL (range 0.18-15.34 ng/mL). High sensitivity Troponin I was reported in 6% of patients and the median of highest reported values was 6.90 ng/mL (range 6.77-14.35 ng/mL). The remaining 18.8% of patients were reported to have had an elevated troponin but analysis could not be performed due to lack of specific details.

Inflammatory biomarkers: 75% of patients included reported elevation of inflammatory biomarkers. 92% of patients had an elevated CRP, and 23% of patients had an elevation of ESR during the evaluation of myocarditis. Other biomarkers like BNP (15%), D-dimer (7.7%), and CPK (5.7%) were also noted to be elevated in some of the patients.

Electrocardiogram: EKG findings were reported in 95.6% of patients. 78.8% of patients had ST-elevation, 7.5% had non-specific ST-T changes, and 7.5% of patients had no significant EKG changes. PR depression, ST depression, and peak T waves were rarely reported (1.5%). One patient presented with AV dissociation with junctional rhythm along with ST-elevation.

Transthoracic echocardiogram: Echocardiogram findings were reported in 80% of the patients. 67.3 % of the patients had an ejection fraction of more than 50% with no regional wall motion abnormalities. 29.1% of patients reported hypokinesis/regional wall motion abnormalities on echocardiographic findings. Decreased EF of less than 50% was reported in 9.1% of patients. Pericardial effusion was noted in only 3.6% of the patients.

Cardiac MRI: Cardiac MRI findings were reported in 87% of the patients. The myocarditis was confirmed on MRI in all the patients, with late gadolinium enhancement being reported in 88.3% of the patients, indicating myocardial necrosis/fibrosis.

Treatment: Specific anti-inflammatory therapy was administered in 60.9% of the patients. 85.7% of patients received NSAIDs, 50% patients received colchicine, 21.4% patients received steroids, and 14.3% patients received IVIG.

Prognosis: Outcomes were reported in 87% of the patients. All the reported patients have recovered from acute myocarditis and were discharged from the hospital. Hospital length of stay was reported in 59.4% of the patients. Median LOS was four days (range 2-9 days). The results are summarized in Table 2.

Discussion

Epidemiology

**Figure 2:**
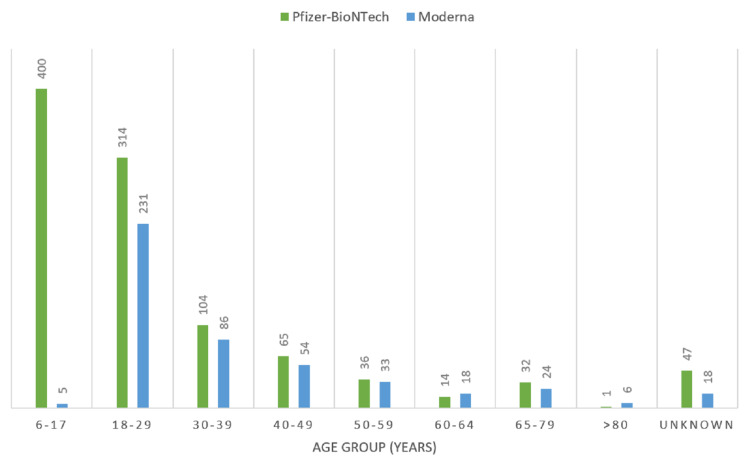
Incidence of post-mRNA vaccination myocarditis by age groups as per CDC VAERS data accessed on September 2, 2021. Number of cases represented by bars. CDC: Centers for Disease Control, VAERS: Vaccine Adverse Event Reporting System.

**Figure 3:**
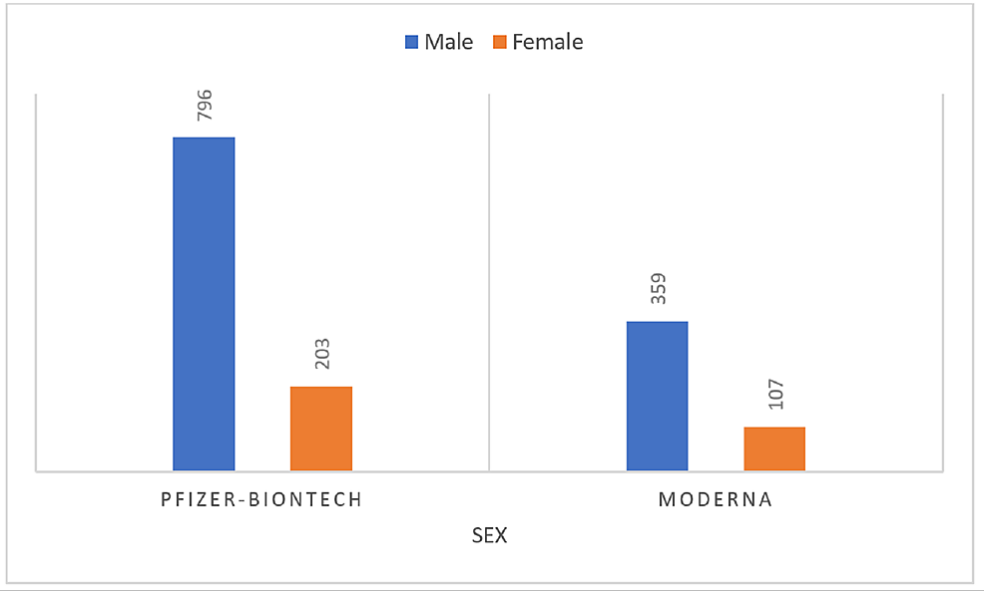
Incidence of post-mRNA vaccination myocarditis by sex as per CDC VAERS data accessed on September 2, 2021 Number of cases represented by bars. CDC: Centers for Disease Control, VAERS: Vaccine Adverse Event Reporting System.

The CDC VAERS data showed a high incidence of post-mRNA vaccination myocarditis in males less than 30 years of age (Figures 2-3) [5].

Our pooled data also showed that the incidence of myocarditis post mRNA vaccination is predominantly seen in young males. Most of the patients present within a few days of vaccination with chest pain being the predominant symptom. Most patients had ST-elevation on EKG and MRI was diagnostic in all the patients who underwent the imaging. All the patients with outcomes reported have recovered. Dickey et al. reported a case series of six male patients with an age range of 17-37 years [29]. All six patients presented with chest pain and elevated troponin. Five of them had an ST elevation on EKG. Myocarditis was confirmed on MRI in the four patients who underwent imaging and all of them recovered [29]. Montgomery et al. also reported a case series of 23 male patients with a median age of 25 years (range 20-51 years) [16]. All the patients presented with chest pain and elevated troponin. Cardiac MRI was abnormal in the eight patients who underwent imaging. All the patients recovered [16]. The patient characteristics of both the case series reflect the findings we noticed in our pooled analysis.

Etiopathogenesis

The pathophysiology of the post-mRNA vaccine myocarditis is currently unknown given the recent identification of the condition and the limited cases reported in the literature. Histopathology has not been reported yet as only two patients in the data underwent myocardial biopsy, but they did not show any significant pathology [10,21]. Given the significant incidence of myocarditis after the second dose, it could be postulated that the pathology could be hypersensitivity myocarditis [16], with the first dose of the vaccine being the sensitizing dose. Our study also showed that among patients who had a previous infection with COVID-19, three out of six patients developed myocarditis following their first dose of vaccination. Montgomery et al. also reported three patients who developed myocarditis after the first dose of vaccination and all three had a history of confirmed COVID-19 infection [16]. In these patients, the infection could have been the sensitizing event and lends weight to the probability of hypersensitivity myocarditis. The original study by Baden et al. on the Moderna vaccine reported side effects like injection site pain, erythema, induration, lymphadenopathy, and systemic events like fever, chills, fatigue, myalgia, arthralgia, nausea, and vomiting which were more common in younger participants (18-65 years) [2]. Similarly, the study by Polack et al. on the Pfizer-BioNTech vaccine reported local reactions like injection site pain, swelling and redness, and systemic events like fever, fatigue, headache, lymphadenopathy which also were more common in younger individuals (16-55 years) [3]. In the initial clinical trials of the mRNA vaccines, systemic adverse events were noted to be more in younger adults probably due to increased immunogenicity in younger individuals [2,3]. That could potentially explain the significantly high incidence of post-vaccine myocarditis seen in younger individuals [5].

Muthukumar et al. reported one case and performed an in-depth study into the potential pathological mechanism [20]. Previous COVID-19 infection in the patient was ruled out by testing for antibodies against 18 different COVID-19 antigens. Serological testing and respiratory PCR also ruled out active infection with Epstein Barr virus (EBV), Cytomegalovirus (CMV), respiratory pathogen panel, Mycoplasma, and Coxsackie B virus. The patient was tested for 121 known genes potentially linked to cardiomyopathy and pathological variants were identified. Cytokine analysis showed elevated levels of Interleukins IL-1ra, IL-5, IL-16, and Monokine induced by gamma (MIG). But inferences cannot be drawn from these data because the data are from only one patient. A few autoantibodies were seen by their significance is unknown [20]. Other authors have also tested for active viral infections including influenza, common respiratory pathogen panel, EBV, CMV, HIV, hepatitis B, C, Coxiella, adenovirus, enterovirus, parvovirus B19, and Mycoplasma without any positive results [1,7,8,10,12,14-19,23-29,31]. Other proposed mechanisms include molecular mimicry between SARS-COV-2 spike protein and self-antigens and auto-antibody generation leading to myocardial damage [33].

Cardiac MRI

Early features of myocardial inflammation on a cardiac MRI include myocardial edema and hyperemia [34]. Late gadolinium enhancement (LGE) represents permanent damage to the myocardium, necrosis in the acute state, and scarring in the chronic state. The areas of LGE may appear larger in the acute setting since they are accompanied by edema and may decrease in size or disappear in the long run due to scar shrinkage [34]. Late gadolinium enhancement is a prognostic factor for increased cardiac mortality and sudden cardiac death in a study on follow-up patients with viral myocarditis [35]. Since the cardiac MRIs of patients in the analysis were obtained in the acute setting, long-term follow-up is needed to understand the degree of permanent scarring in the myocardium and prognosis. The persistence of LGE and disappearance of edema indicate replacement fibrosis and are markers of unfavorable prognosis [36].

Management and Outcome

Endomyocardial biopsy may not be useful because of the often patchy nature of myocarditis [37]. There are no current uniform guidelines regarding management. NSAIDs, colchicine, steroids, and IVIG have been used by different authors. Further studies are needed in the area. Patients need to be reassured that the condition has a good prognosis, as all the reported patients recovered and were discharged home. The expert opinion is to restrict strenuous physical activity for at least three to six months after the episode of myocarditis [38].

Strengths and Limitations

The strength of this article is that, to the best of our knowledge, this is the largest pooled analysis of individual data of 69 patients from 25 case series and case reports of post mRNA vaccination myocarditis. We also reviewed two articles reporting 29 more patients which showed similar findings to ours but were not included in the pooled analysis. Since the VAERS is not an active surveillance system, not all cases of myocarditis may be reported. Mild cases of post-vaccination myocarditis may not be admitted to the hospital. Not all clinical cases are reported in the literature. The reported cases may not include all the pertinent clinical data of their patients. The underlying pathophysiology of the condition has not been identified yet. Due to the recent nature of the condition, the long-term prognosis is unknown. Since the mRNA vaccine is only available in a few countries, the incidence of myocarditis across diverse geographical areas and populations is unknown.

## Conclusions

Post-mRNA vaccination myocarditis is seen predominantly in young males with a median age of 21 years. Most cases present within a few days following the second dose of vaccination. Chest pain is the predominant symptom followed by fever and myalgia. Abnormal EKG is seen in most patients. Laboratory anomalies include elevated troponin and inflammatory markers. Late gadolinium enhancement representing myocardial necrosis or fibrosis can be seen in most patients on cardiac MRI. The diagnosis is made by typical presentation with chest pain with a recent history of mRNA vaccination, an elevated troponin, and characteristic findings on cardiac MRI. The prognosis is good as all the reported patients recovered and were discharged from the hospital. Further research is needed to identify the underlying pathophysiology of myocarditis post-vaccination and to delineate the standard of care.
